# Prognostic model for psychological outcomes in ambulatory surgery patients: A prospective study using a structural equation modeling framework

**DOI:** 10.1371/journal.pone.0193441

**Published:** 2018-04-11

**Authors:** Hendrik-Jan Mijderwijk, Robert Jan Stolker, Hugo J. Duivenvoorden, Markus Klimek, Ewout W. Steyerberg

**Affiliations:** 1 Department of Anesthesiology, Erasmus University Medical Center, Rotterdam, The Netherlands; 2 Department of Public Health, Erasmus University Medical Center, Rotterdam, The Netherlands; Department of Psychiatry and Neuropsychology, Maastricht University Medical Center, NETHERLANDS

## Abstract

**Introduction:**

Surgical procedures are increasingly carried out in a day-case setting. Along with this increase, psychological outcomes have become prominent. The objective was to evaluate prospectively the prognostic effects of sociodemographic, medical, and psychological variables assessed before day-case surgery on psychological outcomes after surgery.

**Methods:**

The study was carried out between October 2010 and September 2011. We analyzed 398 mixed patients, from a randomized controlled trial, undergoing day-case surgery at a university medical center. Structural equation modeling was used to jointly study presurgical prognostic variables relating to sociodemographics (age, sex, nationality, marital status, having children, religion, educational level, employment), medical status (BMI, heart rate), and psychological status associated with anxiety (State-Trait Anxiety Inventory (STAI), Hospital Anxiety and Depression Scale (HADS-A)), fatigue (Multidimensional Fatigue Inventory (MFI)), aggression (State-Trait Anger Scale (STAS)), depressive moods (HADS-D), self-esteem, and self-efficacy. We studied psychological outcomes on day 7 after surgery, including anxiety, fatigue, depressive moods, and aggression regulation.

**Results:**

The final prognostic model comprised the following variables: anxiety (STAI, HADS-A), fatigue (MFI), depression (HADS-D), aggression (STAS), self-efficacy, sex, and having children. The corresponding psychological variables as assessed at baseline were prominent (i.e. standardized regression coefficients ≥ 0.20), with STAI-Trait score being the strongest predictor overall. STAI-State (adjusted R^2^ = 0.44), STAI-Trait (0.66), HADS-A (0.45) and STAS-Trait (0.54) were best predicted.

**Conclusion:**

We provide a prognostic model that adequately predicts multiple postoperative outcomes in day-case surgery. Consequently, this enables timely identification of vulnerable patients who may require additional medical or psychological preventive treatment or–in a worst-case scenario–could be unselected for day-case surgery.

## Introduction

Surgical procedures are increasingly carried out in a day-case setting [1, 2]. The patients’ perception of perioperative health in day-case surgery is currently not dominated by medical factors but by psychological factors [3, 4], including anxiety, depressive moods, aggression, and feelings of fatigue [5]. This situation calls for new research to identify predictive factors for these clinical psychological outcomes to aid early clinical decision making, a task that particularly falls to anesthesiologists in preoperative assessment [6]. Predicting psychological outcomes after day-case surgery is important because poor outcomes could lead to negative socioeconomic effects due to prolonged convalescence that delays a return to normal activities and work [7–11]. Furthermore, an estimated 80% of elective surgical procedures will be carried out as day-case surgery [12], a number that is likely to increase because more and more complex surgery (e.g., craniotomies for brain tumor resection) are carried out in this setting [13, 14]. Accordingly, the probability that patients will experience poor psychological outcomes will increase, underlining the need for adequate prognostic models tailored to predict multiple psychological outcomes after surgery to facilitate prevention.

Prognostic models are statistical models that combine data from patients to predict clinical outcome [15]. Such models based on data collected soon after presentation could in theory be used to aid early clinical decision making and allow for more accurate counseling of patients [15]. Conventionally, prognostic studies aim to find prognostic factors that accurately predict a single outcome variable [16]. However, joint prediction of interrelated outcome variables, such as psychological outcome variables, has not yet been studied extensively. To that end, advanced statistical methodology like Structural Equation Modeling (SEM) is needed. SEM enables joint analyses of several candidate prognostic factors on several outcome variables [17]. SEM has been used in many research fields and is currently coming into use in clinical medicine [18]. In the field of anesthesiology, however, it has been rarely used, although there have been calls to further establish use of this statistical methodology [19].

We aimed to develop a prognostic model based on sociodemographic, medical, and psychological variables assessed just before day-case surgery on psychological outcomes after surgery using SEM. This model could help to preserve the medical and socioeconomic success of surgery in a day-case setting.

## Methods

### Study population and study design

**Fig 1:**
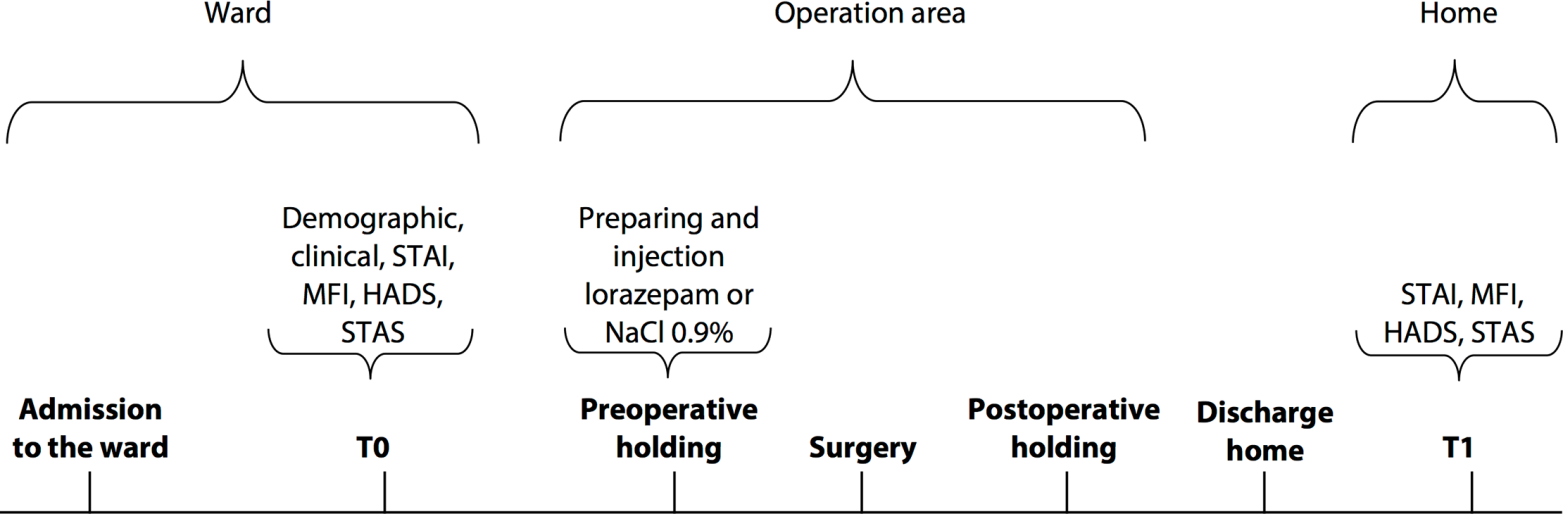
Timeline of the study. T0 = baseline assessment on the day of surgery (self-reported questionnaire); T1 = seventh postoperative day (self-reported questionnaire); STAI, State-Trait Anxiety Inventory; MFI, Multidimensional Fatigue Inventory; HADS, Hospital Anxiety and Depression Scale, STAS: State-Trait Anger Scale.

This study is part of a larger double-blinded randomized controlled clinical trial conducted at the Erasmus University Medical Center, comparing the effects of lorazepam and placebo in day-case surgery patients [5]. However, the methods in this study have been adapted to address different objectives. We recruited patients from our day-case surgery department between October 2010 and September 2011. We included all patients who were referred for day-case surgery and aged at least 18 years. Patients were excluded if they met one or more of the following criteria: insufficient command of the Dutch language; severe learning difficulties; or undergoing ophthalmology surgery, extracorporeal shock wave lithotripsy, endoscopy, Botox treatment, abortion, or chronic pain treatment. The latter procedures are generally considered to be minimally invasive. Most practitioners are of the opinion that these procedures do not require premedication. Finally, prior use of psychopharmaceuticals and contraindication to lorazepam use–according to our national pharmacotherapeutic compass (available at http://www.farmacotherapeutischkompas.nl; accessed 2010)–were also exclusion criteria. The study protocol was approved by the Medical Ethical Committee of Erasmus MC (Chairperson Prof. dr. H.W. Tilanus) and by the Netherlands Central Committee on Research involving Human Subjects (CCMO) and registered with EudraCT under number 2010-020332-19. The trial has also been registered under identification number NCT01441843 in the ClinicalTrials.gov protocol registration system. Written informed consent was obtained from all participants. The time schedule for the current study is shown in Fig 1.

### Outcome variables

#### State-Trait Anxiety Inventory (STAI)

Anxiety was measured using the Dutch version of the STAI [20]. The STAI consists of two 20-item scales. One scale measures how one feels in general (Trait anxiety) while the other measures how one feels at the present moment (State anxiety). Sum scores for both scales were calculated by adding the scores of all the items, ranging from 20 to 80. A higher score indicates a higher level of anxiety. STAI has good validity, and the STAI-State and STAI-Trait scales have similar reliability scores [20].

#### Multidimensional Fatigue Inventory (MFI)

Fatigue was measured using the Dutch version of the MFI [21], a 20-item questionnaire covering five scales: General fatigue, Physical fatigue, Mental fatigue, Reduced motivation, and Reduced activity. A sum score was calculated by adding the scores of all the items, ranging from 20 to 100. A higher score indicates a higher degree of fatigue. In the majority of cases, MFI has good validity and reliability [21].

#### Hospital Anxiety and Depression Scale (HADS)

Depressive moods were measured using a Dutch version of the HADS [22], which consists of two 7-item scales: one for anxiety (HADS-A) and one for depression (HADS-D). Each item comprises four answer alternatives, and for each of the scales the total score ranges from 0 to 21. A higher score indicates a higher degree of either anxiety or depression. The HADS has adequate validity and internal consistency in the Dutch population [23].

#### State-Trait Anger Scale (STAS)

Aggression regulation was assessed using the Dutch version of the STAS [24], which consists of two 10-item scales, one covering the State-aggression (how angry one feels at the moment) and one covering the Trait-aggression (how angry one feels in general). The sum scores range from 10 to 40. A higher score indicates a higher degree of aggression. Both subscales have adequate validity and reliability [24].

#### Sociodemographic and medical prognostic variables

The sociodemographic variables we considered were sex, age, educational level, marital status, employment, religion, having children, and type of nationality (i.e. Dutch versus non-Dutch). The medical variables we considered were Body Mass Index (BMI) and preoperative heart rate (HR).

### Psychological prognostic variables

#### Baseline assessment of outcome variable

Baseline assessments of all psychological outcome variables were used as candidate prognostic variables.

#### Rosenberg Self-Esteem Scale (RSES)

Self-esteem was measured using the 10-item Dutch version of the RSES [25]. The sum score ranges from 10 to 40. A higher score indicates a higher degree of self-esteem. RSES has good validity and reliability [25].

#### General Self-Efficacy Scale (GSES)

Self-efficacy was measured using the Dutch version of the GSES [26]. The sum score ranges from 10 to 40. A higher score indicates a higher degree of self-efficacy. In addition to an adequate validity, GSES also has an adequate reliability in the Dutch population [27].

#### Statistical analysis

We explored the relations between baseline assessments (T0, just before surgery) and outcome variables (T1, seventh day after surgery). We included sociodemographic, medical, and psychological variables assessed at baseline in the model simultaneously. Predictor variables were entered for all outcomes in the model to allow for insight into the relative importance of each predictor. The analyses were guided by statistical and clinical–theoretical criteria. The first step was to analyze all predictor variables together with the seven outcome variables that were assessed on the seventh day after surgery. In the second step, we eliminated less-relevant predictor variables according to the backward elimination procedure (P-to-remove > 0.20 on at least four outcome variables) provided that there was no substantial loss of information (i.e., a decrease in P value for model fit ≥ 0.10). Type of intervention as randomized was adjusted for.

Modeling was performed using the Maximum Likelihood (ML) an estimation method. The distributions of the variables were considered non-normal, so the final modeling was performed using the Maximum Likelihood for Robustness (MLR) as an estimation method.

The following measures were used to test for adequacy of the model fit:

1. Chi-square for model fit (low and non-significant values of the chi-square are desired; P value > 0.05); 2. Chi-square/degrees of freedom ratio (a value < 2.0 was considered to be acceptable); 3. Comparative Fit Index (CFI) and Tucker-Lewis Index (TLI) (high values are desired (> 0.95) [28, 29]; 4. Root Mean Square Error of Approximation (RMSEA: a value < 0.05 indicates a close fit) [30]; and 5. Standardized Root Mean Squares of Residuals (SRMR: a value of < 0.08 indicates a reliable fit) [31]. After testing for goodness-of-fit, it was of particular clinical interest to calculate the percentages of explained variance for each outcome variable.

There was little missing data (< 5% for all variables), which were not included in the prognostic analysis. Bootstrapping was used for internal validation [32, 33].

Each subsample was a random sample with replacement from the full sample, and we checked the internal validity of the prognostic model using 1000 bootstrap samples. Bias-corrected standard errors were estimated. For each predictor, we estimated the unstandardized regression coefficients, including the 95% bootstrapped confidence intervals, and standardized regression coefficients as effect estimates.

We used SPSS version 20.0 (IBM Corp. Armonk, NY) and Mplus version 7 (Muthén and Muthén, Los Angeles, CA) for statistical analyses. Estimates were regarded as statistically significant if the two-sided P value was < 0.05.

## Results

We included 398 patients, who all completed measurement at baseline while 383 (96%) completed measurement at follow-up. The study population had more males (56%) than females, and most of the patients were Dutch (94%). The majority (60%) lived together with a partner, and approximately half of the patients had children. About two thirds reported being non-religious. A total of 270 patients (68%) had a middle level of education whereas lower but similar numbers of patients had low (*n* = 63) and high (*n* = 65) levels of education. Three quarters of the patients were employed. The median age was 36.7 years, the median BMI ((body weight in kilograms)/(body height in meters)^2^) was 24.6, and the median preoperative HR (beats per minute) was 69 (Table 1).

**Table 1:** General characteristics of patients at baseline.

***Categorical***			*n*	%
Type of intervention				
Verum (lorazepam)			198	49.7
Placebo (NaCl 0.9%)			200	50.3
Sex				
Female			174	43.7
Male			224	56.3
Nationality				
Dutch			374	94.0
Non-Dutch			24	6.0
Marital status^a^				
Single			158	39.7
Together			240	60.3
Children				
Yes			206	51.8
No			192	48.2
Religion				
Yes			128	32.2
No			270	67.8
Educational level^b^				
Low			63	15.8
Middle-level			270	67.8
High			65	16.3
Employment				
Yes			301	75.6
No			97	24.4
***Continuous***	*n*	Percentiles			
		25	50	75
Age	398	28.8	36.7	49.4
BMI^c^	398	22.4	24.6	27.7
Heart rate^d^	396	62.0	69.0	78.0

^a^ Single: unmarried, divorced, widow(er); Together: married, living together

^b^ Low: no education, elementary school, preparatory middle-level vocational education; Middle-level: middle-level vocational education, higher general continued education, higher vocational education; High: preparatory university education, university education

^c^ Body Mass Index: body weight in kilograms)/(body height in meters)^2^

^d^ Heart rate: beats per minute.

Mean anxiety scores (STAI-State, STAI-Trait and HADS-A) decreased after surgery whereas the mean values for aggression scores (STAS-State and STAS-Trait) and depression scores (HADS-D) remained about the same over time (Table 2). Mean fatigue scores (MFI) increased postoperatively. The differences across time were not tested for statistical significance.

**Table 2:** Descriptive psychological variables.

	Baseline (T0)		7^th^ day after surgery (T1)	
	mean	SD	mean	SD
STAI-State	38.1	9.4	30.3	8.9
STAI-Trait	33.5	8.1	30.5	8.7
HADS-A	4.7	3.1	2.9	2.9
MFI	41.6	13.1	48.5	17.0
STAS-State	10.2	1.2	10.6	2.5
STAS-Trait	13.4	3.6	13.1	3.6
HADS-D	3.0	2.4	2.8	2.9
RSES	33.5	4.4	NA	NA
GSES	31.6	4.2	NA	NA

Abbreviations: STAI-State, State-Trait Anxiety Inventory, State part; STAI-Trait, State-Trait Anxiety Inventory, Trait part; MFI, Multidimensional Fatigue Inventory; HADS-A, Hospital Anxiety and Depression Scale, Anxiety part; HADS-D, Hospital Anxiety and Depression Scale, Depression part; STAS-State, State-Trait Anger Scale, State part; STAS-Trait, State-Trait Anger Scale, Trait part; RSES, Rosenberg Self-Esteem Scale; GSES, General Self-Efficacy Scale; NA, not applicable. The differences across time were not tested for statistical significance. T0: *n* = 398; T1: *n* = 383.

### Correlations between prognostic variables and outcomes over time

At T0 (baseline), the highest correlations were found between HADS-A and STAI-Trait (r = 0.66) and HADS-A and STAI-State (r = 0.66; Table 3). Also, STAI-Trait correlated substantially with STAI-State (r = 0.52), MFI (r = 0.54) and HADS-D (r = 0.55). At T1 (seventh day after surgery), the intercorrelations were substantial. The highest correlations were found between STAI-State and STAI-Trait (r = 0.76) and between STAI-State and HADS-A (r = 0.71). STAI-Trait had a correlation of 0.71 with HADS-A. The intracorrelations over time of most of the psychological outcome variables varied from moderate to substantial: STAI-State (r = 0.42), STAI-Trait (r = 0.79), HADS-A (r = 0.59), MFI (r = 0.54), STAS-Trait (r = 0.68), and HADS-D (r = 0.55). STAS-State showed a correlation of only 0.16 (Table 3).

**Table 3:** Correlation matrix of baseline and outcome variables according to the final prognostic model.

	***Baseline predictors (T0)***		1	2	3	4	5	6	7	8	9	10	11	12	13	14	15	16	17
1	Sex																		
2	Children		-0.03																
3	Anxiety	STAI-State	**0.27**	-0.03															
4		STAI-Trait	**0.16**	-0.07	**0.52**														
5		HADS-A	**0.18**	-0.05	**0.66**	**0.66**													
6	Fatigue	MFI	**0.12**	-0.07	**0.38**	**0.54**	**0.42**												
7	Aggression	STAS-State	-0.02	-0.08	**0.13**	**0.15**	**0.11**	0.02											
8		STAS-Trait	0.05	-0.05	**0.15**	**0.47**	**0.27**	**0.23**	**0.25**										
9	Depression	HADS-D	-0.02	0.05	**0.35**	**0.55**	**0.48**	**0.49**	0.00	**0.20**									
10	Self-efficacy	GSES	**-0.16**	0.09	**-0.24**	**-0.32**	**-0.28**	**-0.27**	0.08	**-0.10**	**-0.23**								
	***Outcomes (T1)***																		
11	Anxiety	STAI-State	**0.11**	0.04	**0.42**	**0.61**	**0.51**	**0.51**	**0.13**	**0.28**	**0.46**	**-0.14**							
12		STAI-Trait	**0.12**	-0.03	**0.45**	**0.79**	**0.63**	**0.52**	**0.16**	**0.43**	**0.55**	**-0.27**	**0.76**						
13		HADS-A	0.06	-0.02	**0.35**	**0.59**	**0.59**	**0.40**	**0.14**	**0.30**	**0.47**	**-0.14**	**0.71**	**0.71**					
14	Fatigue	MFI	**0.17**	0.03	**0.29**	**0.34**	**0.31**	**0.54**	0.04	**0.16**	**0.30**	**-0.11**	**0.58**	**0.45**	**0.46**				
15	Aggression	STAS-State	0.07	0.07	**0.15**	**0.34**	**0.31**	**0.21**	**0.16**	**0.26**	**0.18**	-0.08	**0.51**	**0.44**	**0.52**	**0.30**			
16		STAS-Trait	**0.13**	0.00	**0.25**	**0.54**	**0.41**	**0.25**	0.09	**0.68**	**0.25**	**-0.15**	**0.45**	**0.56**	**0.54**	**0.26**	**0.54**		
17	Depression	HADS-D	0.04	0.09	**0.26**	**0.52**	**0.36**	**0.43**	0.09	**0.28**	**0.55**	**-0.16**	**0.66**	**0.60**	**0.63**	**0.57**	**0.45**	**0.40**	

Abbreviations: STAI-State, State-Trait Anxiety Inventory, State part; STAI-Trait, State-Trait Anxiety Inventory, Trait part; MFI, Multidimensional Fatigue Inventory; HADS-A, Hospital Anxiety and Depression Scale, Anxiety part; HADS-D, Hospital Anxiety and Depression Scale, Depression part; STAS-State, State-Trait Anger Scale, State part; STAS-Trait, State-Trait Anger Scale, Trait part; GSES, General Self-Efficacy Scale. Significant values (P <0.05; two-tailed) are represented in bold; Sex: 0 = male, 1 = female; Children: 0 = no children, 1 = having children; T1 = 7th day after surgery. Gray: intracorrelations of psychological variables.

### Prognostic potentialities of baseline variables

The final model comprised the following predictors: sex, having children, STAI-State, STAI-Trait, HADS-A, MFI, STAS-State, STAS-Trait, HADS-D, and GSES. Nationality, marital status, religion, educational level, employment, age, BMI, HR, RSES and type of intervention as randomized were fixed at zero. The performance measures all showed adequate values: using the MLR as the estimation method, the P value for the chi-square for model fit (98.99; df = 77) turned out to be just significant (P = 0.05) while the ML estimation method yielded a chi-square value of 97.36 (df = 77; P = 0.06). The chi-square/degrees of freedom ratio was 1.29. The comparative fit index was 0.99, and the Tucker–Lewis Index was 0.98. RMSEA was 0.03 (90% confidence interval: 0.004 to 0.042) and the SRMR was 0.02. The demographic variables had minor effects in the final model, and adding the medical variables did not affect the performance of the final model. In contrast, the prognostic potential of psychological baseline measurements was substantial. The adjusted explained variances (R^2^ adjusted) ranged from 0.15 for State aggression (STAS-State) to 0.66 for Trait anxiety (STAI-Trait). STAI-State, HADS-A, MFI, STAS-Trait and HADS-D showed R^2^ adjusted scores of 0.44, 0.45, 0.31, 0.54 and 0.38, respectively. In all, with the exception of aggression, the outcome variables were substantially predictable (Table 4).

**Table 4:** Prognostic performance of the baseline variables in the final model: Explained variances.

		R^2^ Adjusted		
		A	B	C
*Outcome variables*				
Anxiety	STAI-State	0.01	0.01	0.44
	STAI-Trait	0.01	0.01	0.66
	HADS-A	0.00	0.00	0.45
Fatigue	MFI	0.02	0.02	0.31
Aggression	STAS-State	0.01	0.01	0.15
	STAS-Trait	0.01	0.01	0.54
Depression	HADS-D	0.00	0.00	0.38

Abbreviations: STAI-State, State-Trait Anxiety Inventory, State part; STAI-Trait, State-Trait Anxiety Inventory, Trait part; MFI, Multidimensional Fatigue Inventory; HADS-A, Hospital Anxiety and Depression Scale, Anxiety part; HADS-D, Hospital Anxiety and Depression Scale, Depression part; STAS-State, State-Trait Anger Scale, State part; STAS-Trait, State-Trait Anger Scale, Trait part; GSES, General Self-Efficacy Scale.

A = Demographic variables assessed at baseline.

B = Demographic and medical variables assessed at baseline.

C = Demographic, medical, psychological variables assessed at baseline.

**Fig 2:**
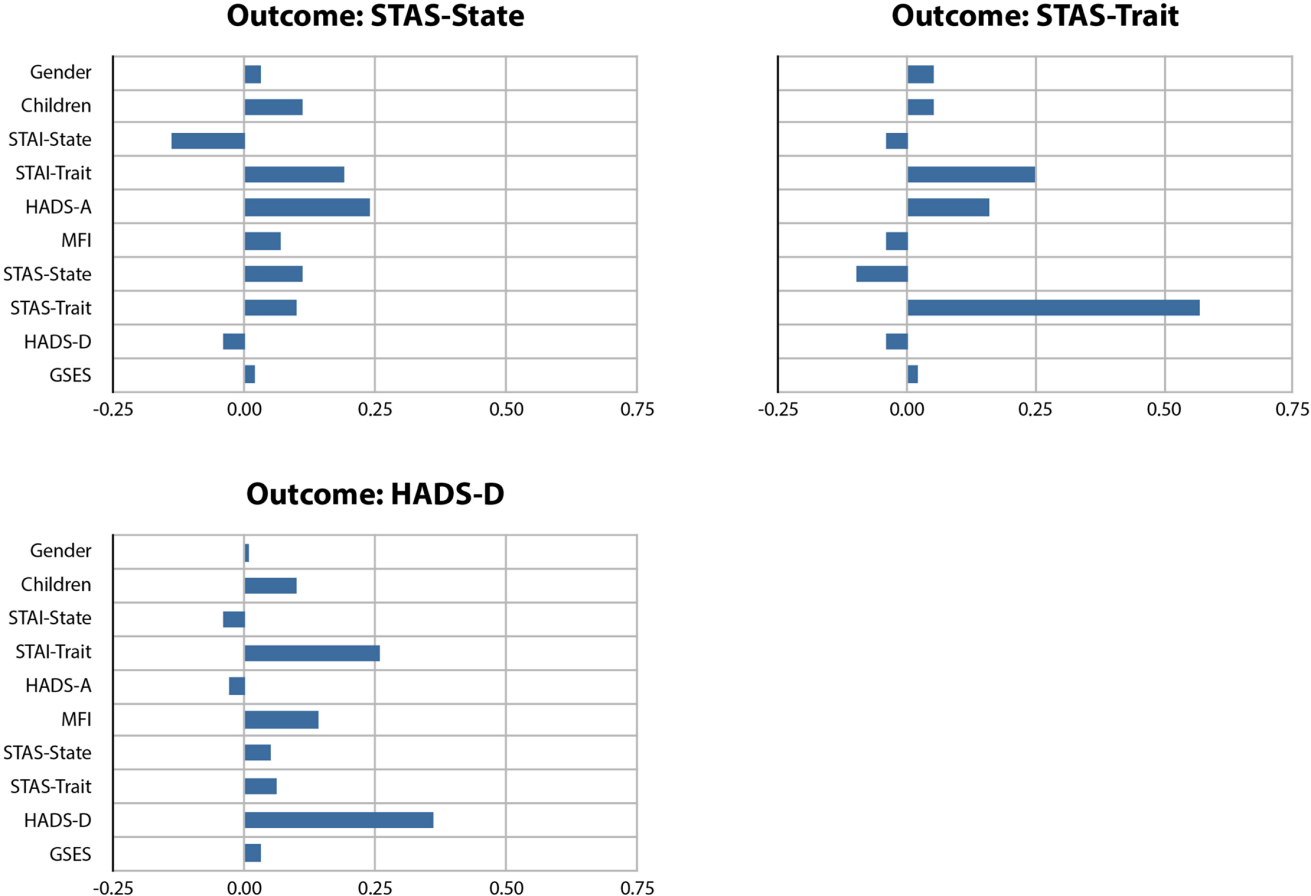
Prognostic potentialities of the final predictor variables distinguished by outcome variables in a day-case surgery population.

For each outcome variable, on the seventh day after surgery (T1), we considered the important prognostic variables according to the standardized estimates (B), as shown in Tables 5 and 6. We focused on standardized estimates with a value of ≥ 0.20 only (Fig 2).

**Table 5:** Individual estimates of the final prognostic model (I/II).

		Anxiety (STAI-State)				Anxiety (STAI-Trait)				Anxiety (HADS-A)			
		b^#^	95% CI_b_		B^$^	b	95% CI_b_		B	b	95% CI_b_		B
Intercept		-4.48	-12.90	5.11	NA	0.88	-5.66	8.22	NA	-5.41	-8.35	-2.23	NA
Sex		0.20	-1.22	1.50	0.01	0.00	-1.09	1.07	0.00	-0.12	-0.61	0.34	-0.02
Children		1.48	0.07	2.76	0.08	0.42	-0.63	1.44	0.02	0.09	-0.40	0.51	0.02
Anxiety	STAI-State	0.04	-0.06	0.14	0.05	-0.04	-0.12	0.04	-0.05	-0.04	-0.07	-0.01	-0.13
	STAI-Trait	0.40	0.22	0.55	**0.36**	0.56	0.44	0.67	**0.52**	0.10	-0.05	0.14	**0.27**
	HADS-A	0.34	-0.08	0.79	0.12	0.50	0.15	0.85	0.18	0.39	0.26	0.51	**0.41**
Fatigue	MFI	0.16	0.09	0.23	**0.23**	0.07	0.02	0.11	0.10	0.02	-0.01	0.04	0.08
Aggression	STAS-State	0.39	-0.57	0.77	0.05	0.35	-0.10	0.88	0.05	0.14	-0.12	0.31	0.06
	STAS-Trait	0.00	-0.26	0.25	0.00	0.20	0.03	0.41	0.08	0.03	-0.06	0.11	0.03
Depression	HADS-D	0.33	-0.09	0.78	0.09	0.46	0.16	0.78	0.13	0.17	0.03	0.31	0.14
Self-Efficacy	GSES	0.19	0.00	0.38	0.09	-0.01	-0.15	0.13	-0.01	0.06	0.00	0.11	0.08

Abbreviations: STAI-State, State-Trait Anxiety Inventory, State part; STAI-Trait, State-Trait Anxiety Inventory, Trait part; MFI, Multidimensional Fatigue Inventory; HADS-A, Hospital Anxiety and Depression Scale, Anxiety part; HADS-D, Hospital Anxiety and Depression Scale, Depression part; STAS-State, State-Trait Anger Scale, State part; STAS-Trait, State-Trait Anger Scale, Trait part; GSES, General Self-Efficacy Scale; NA, not applicable.

^#)^b = unstandardized regression estimate

^$)^B = standardized regression estimate; CI_b_ = bootstrapped confidence interval for corresponding b; Sex: 0 = male, 1 = female; Children: 0 = no children, 1 = having children. Used method: ML estimation.

**Table 6:** Individual estimates of the final prognostic model (II/II).

		Fatigue (MFI)				Aggression State (STAS-State)				Aggression Trait (STAS-Trait)				Depression (HADS-D)			
		b	95% CI_b_		B	b	95% CI_b_		B	b	95% CI_b_		B	b	95% CI_b_		B
Intercept		2.76	-15.91	22.83	NA	4.88	1.63	9.15	NA	4.10	0.58	8.12	NA	-5.14	-8.59	-1.75	NA
Sex		3.50	0.51	6.48	0.10	0.15	-0.21	0.61	0.03	0.36	-0.07	0.88	0.05	0.04	-0.43	0.52	0.01
Children		2.48	-0.55	5.20	0.07	0.56	0.18	1.11	0.11	0.35	-0.14	0.88	0.05	0.58	0.13	1.05	0.10
Anxiety	STAI-State	0.06	-0.16	0.26	0.04	-0.04	-0.07	-0.01	-0.14	-0.02	-0.05	0.02	-0.04	-0.01	-0.05	0.02	-0.04
	STAI-Trait	-0.02	-0.32	0.29	-0.01	0.06	0.00	0.13	0.19	0.11	0.05	0.19	**0.25**	0.09	0.04	0.15	**0.26**
	HADS-A	0.39	-0.42	1.07	0.07	0.19	0.04	0.37	**0.24**	0.19	0.05	0.37	0.16	-0.02	-0.13	0.11	-0.03
Fatigue	MFI	0.65	0.50	0.80	**0.50**	0.01	-0.01	0.04	0.07	-0.01	-0.04	0.01	-0.04	0.03	0.01	0.06	0.14
Aggression	STAS-State	0.23	-0.67	1.79	0.02	0.21	-0.15	0.44	0.11	-0.28	-0.62	-0.02	-0.10	0.12	-0.06	0.39	0.05
	STAS-Trait	0.07	-0.47	0.62	0.01	0.07	-0.02	0.20	0.10	0.58	0.48	0.68	**0.57**	0.05	-0.04	0.14	0.06
Depression	HADS-D	0.13	-0.70	0.86	0.02	-0.04	-0.20	0.09	-0.04	-0.06	-0.18	0.09	-0.04	0.43	0.28	0.59	**0.36**
Self-efficacy	GSES	0.27	-0.15	0.65	0.07	0.01	-0.06	0.07	0.02	0.01	-0.06	0.08	0.02	0.02	-0.05	0.08	0.03

Abbreviations: STAI-State, State-Trait Anxiety Inventory, State part; STAI-Trait, State-Trait Anxiety Inventory, Trait part; MFI, Multidimensional Fatigue Inventory; HADS-A, Hospital Anxiety and Depression Scale, Anxiety part; HADS-D, Hospital Anxiety and Depression Scale, Depression part; STAS-State, State-Trait Anger Scale, State part; STAS-Trait, State-Trait Anger Scale, Trait part; GSES, General Self-Efficacy Scale; NA, not applicable.

^#)^b = unstandardized regression estimate

^$)^B = standardized regression estimate; CI_b_ = bootstrapped confidence interval for corresponding b; Sex: 0 = male, 1 = female; Children: 0 = no children, 1 = having children. Used method: ML estimation.

For STAI-State, baseline STAI-Trait was the most important predictor (B = 0.36), followed by MFI (B = 0.23). In contrast, the baseline assessment of STAI-State had no high prognostic impact. For STAI-Trait, only baseline STAI-Trait was important (B = 0.52). For HADS-A, baseline HADS-A had the highest prognostic effect (B = 0.41), followed by STAI-Trait (B = 0.27). For MFI, only baseline MFI was of high prognostic relevance (B = 0.50). For STAS-State, HADS-A had substantial prognostic effect (B = 0.24). In contrast, the baseline assessment of STAS-State had no high prognostic impact. For STAS-Trait, baseline STAS-Trait dominated the prognostic variables (B = 0.57). STAI-Trait was also an important prognostic variable (B = 0.25). For HADS-D, two predictor variables were of prognostic importance: HADS-D (B = 0.36) and STAI-Trait (B = 0.26). The residuals of the outcomes were moderately interrelated (intercorrelations between 0.15 and 0.54).

## Discussion

We developed a prognostic model using sociodemographic, medical, and psychological variables assessed just before day-case surgery that predicts multiple psychological outcomes after day-case surgery. Overall, apart from state aggression, the psychological outcome variables could be adequately predicted using the identified prognostic model. Sociodemographic and medical variables were of minor importance, with the exception of sex (females are at higher risk for a poor psychological outcome) and having children. In contrast, the psychological variables as assessed at baseline were of prominent importance.

This model is of interest for improving patients’ quality of recovery and is useful for preoperative decision making. The model can be easily applied in the clinical setting because the parameters can all be completed by patient self-assessment. In the next step, the model results give healthcare professionals the possibility of identifying patients at risk for poor psychological outcomes after the surgical procedure. After identification, patients at risk can be guided in adequate follow-up or–in a worst-case scenario–can be unselected for day-case surgery. We believe that such a prognostic model can be of substantial additional benefit in prehabilitation programs. Recently, prehabilitation programs have showed that optimal preoperative preparation leads to better postoperative outcomes [34], and these programs improve postoperative outcomes by using preoperative interventions. Surgical procedures in day-case surgery are electively planned, so the preoperative period can be used to implement intervention within a prehabilitation program framework. Prehabilitation programs may differ for different surgical populations [35], and should therefore be tailored to the population of interest. One thing that should be developed in every single prehabilitation program is a preoperative prognostic model directed to vulnerable patients [34]. The provided preoperative prognostic model in this study enables simultaneously prediction of multiple outcomes of interest in day-case surgery by means of only one prognostic model. Accordingly, identification and management of patients at risk becomes feasible, which could lead to better postoperative psychological outcomes together with a reduction in negative socioeconomic effects.

The model is also of interest for prognosis-related research dealing with psychological outcomes. First, from a clinical–theoretical perspective, the STAI-Trait questionnaire score showed to be a valuable prognostic variable for almost all psychological outcome variables expect postoperative fatigue (B > 0.20), although it was not powerful enough to replace the model. Sensitivity analysis that included only STAI-Trait as the prognostic variable showed that only anxiety (STAI-State and STAI-Trait) as outcome variable was still adequately predicted, R^2^ equalled 0.37 and 0.62 respectively. Concerning aggression and depression, the reduction in R^2^ was considered too steep, i.e. R^2^ percentage reduction of > 20%. That the STAI-Trait was not an important variable for predicting fatigue (R^2^ < 0.20) is in line with earlier findings [36], but understanding why postoperative fatigue does not follow the mechanisms of other psychological factors requires further study. Christensen et al. have suggested that the mechanisms cannot be explained by psychological factors [37]. More recently, though, it has been postulated that the underlying mechanism should be explained by psychological factors and mainly by its measurement itself [36]. Our results support the latter. Second, from a statistical modeling perspective, here we have applied SEM, a strong statistical approach, to evaluate the joint potentialities of several variables in predicting several outcomes. This joint analysis is especially preferred because psychological outcome variables are likely to interact with one another [38], which also was observed in the present study. Next to this theoretical rationale of an integral analysis, SEM has a couple of additional advantages. It enables achieving a consistent set of predictors for all outcomes and accordingly enables comparison of the regression weights for the different outcome variables, which is difficult and time consuming with a conventional analysis (i.e., predicting each outcome individually). Furthermore, SEM tests if the prediction model adequately represents the data structure, with random fluctuation, and gives insight into the related (residual) intercorrelations of the outcomes. If these residual intercorrelations were to be high for some or all outcome variables, a principal component analysis or a partial least squares regression analysis would be indicated. This information would be missed in case of individual outcome analysis. It has to be noted that ideally, a latent modeling approach seems indicated for analyzing the joint prediction of observed variables. However, we have refrained from using this approach because in clinical practice, it is not plausible to obtain the measurements without error.

### Future considerations

Of the psychological outcomes analyzed, the explained variances (R^2^) were substantial for the anxiety scales (STAI-State, STAI-Trait and HADS-A), the depression scale (HADS-D), the fatigue scale (MFI), and the trait component of aggression regulation (STAS-Trait). However, state aggression (STAS-State) was only moderately predictive. Although we found substantial explained variances as the criterion for assessing the performance of the individual outcomes in the current model, a number of variances still remain unexplained.

This result could arise from the fallibility of the measurements. To assess such fallibility we evaluated the internal consistency of the measurements using Cronbach’s α, both at baseline and at 1 week after surgery. According to the commonly accepted criteria [39], we concluded that internal consistency was satisfactory for all measurements (for the majority of the used scales Cronbach’s α ≥ 0.85, four scales were in the range of 0.73 to 0.79), except for HADS-D assessed at baseline (Cronbach’s α = 0.60). In this study, we used two measurements for anxiety and found the interrelationships of STAI and HADS-A to be non-perfect, in line with previous research [40]. This discrepancy suggests that the different instruments for anxiety have common and unique elements.

In addition, the model might be mis-specified in principle, but we firmly believe that this possibility is not realistic. It is also possible that the phenomenon of omitted variables have played a role in this study. Other unmeasured or as-yet-unknown variables may be relevant and consequently, when added, enhance the prognostic performance of the model. Our study comprises only intrapersonal characteristics, but interpersonal characteristics may also be important. For example, recent research has shown that negative dyadic coping (collaborative coping/dealing with stress within a couple) is associated with a higher degree of psychological distress [41]. Positive dyadic coping seems to be effective in dealing with problems surrounding illness, especially in older couples [42]. Such positive interpersonal variables could also help people cope in a perioperative setting. Environmental variables (e.g., living in suburbs), economic variables (e.g., economic crisis, being unemployed), and cultural variables could also be of interest. Adding such variables to our model may increase its prognostic performance.

### Study limitations and study strengths

First, a limitation of this study was that the assessments were conducted at a single center, which means that further external validation is needed. Second, because we excluded patients who were taking psychopharmaceuticals and those with psychological disorders [5], we can assume that the level of psychological dysfunction after day-case surgery could well be higher. This effect might bias our findings negatively, or strengthen them. Third, the majority of our study population was Dutch, so different results may be obtained when considering broader nationalities or ethnical and sociocultural groups.

Despite these limitations, the fact that our data were obtained in a randomized controlled trial implies their high quality. Another strength of the study is the use of SEM, which appears to be a powerful approach that is suitable for conducting research on optimizing medical decision making using multiple outcome criteria.

## Conclusion

We provide a prognostic model, using a structural equation modeling framework, that adequately predicts multiple outcomes in day-case surgery. The final model comprised the following prognostic variables: anxiety (STAI-State/Trait, HADS-A), fatigue (MFI), depression (HADS-D), aggression (STAS-State/Trait), self-efficacy, sex, and having children. Overall, it enables timely identification of vulnerable patients who may require additional medical or psychological preventive treatment, or–in a worst-case scenario–could be unselected for day-case surgery.

## Supporting information

S1 Data (XLSX)
