# Correction: Prognostic model for psychological outcomes in ambulatory surgery patients: A prospective study using a structural equation modeling framework

**DOI:** 10.1371/journal.pone.0200113

**Published:** 2018-06-28

**Authors:** The PLOS ONE Staff

There is an error in the citation. Author Hendrik-Jan Mijderwijk appears incorrectly. The correct citation is: Mijderwijk H, Stolker RJ, Duivenvoorden HJ, Klimek M, Steyerberg EW (2018) Prognostic model for psychological outcomes in ambulatory surgery patients: A prospective study using a structural equation modeling framework. PLoS ONE 13(4): e0193441. https://doi.org/10.1371/journal.pone.0193441

Fig 2 is incorrect. Please see the corrected Fig 2 here. The publisher apologizes for the errors.

**Fig 2:**
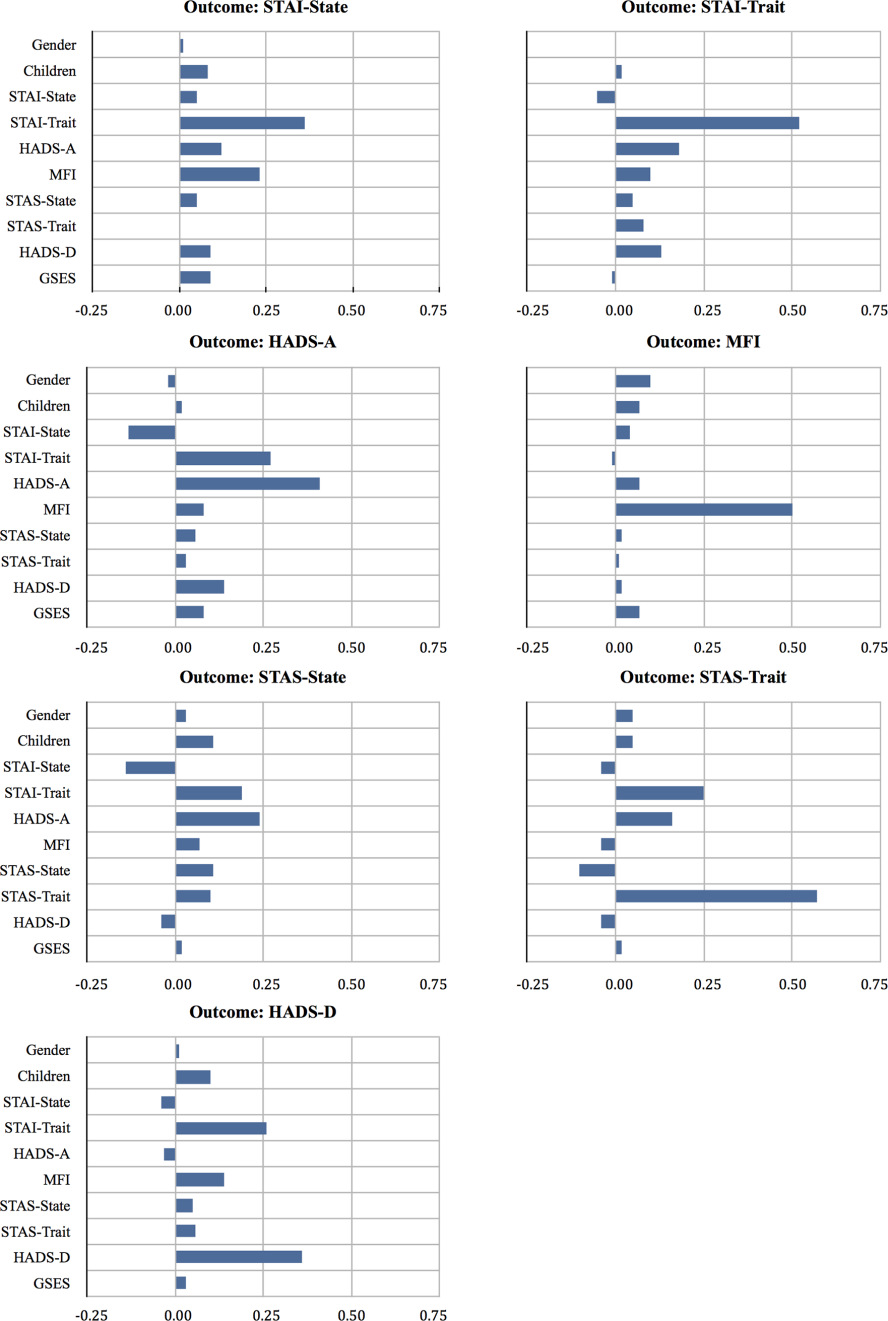
Prognostic potentialities of the final predictor variables distinguished by outcome variables in a day-case surgery population.
